# Multi-Peptide: Multimodality Leveraged Language-Graph Learning of Peptide Properties

**DOI:** 10.1021/acs.jcim.4c01443

**Published:** 2024-12-19

**Authors:** Srivathsan Badrinarayanan, Chakradhar Guntuboina, Parisa Mollaei, Amir Barati Farimani

**Affiliations:** † Department of Chemical Engineering, Carnegie Mellon University, Pittsburgh 15213, Pennsylvania, United States; ‡ Department of Electrical and Computer Engineering, Carnegie Mellon University, Pittsburgh 15213, Pennsylvania, United States; § Department of Mechanical Engineering, Carnegie Mellon University, Pittsburgh 15213, Pennsylvania, United States; ∥ Department of Biomedical Engineering, Carnegie Mellon University, Pittsburgh 15213, Pennsylvania, United States; ⊥ Machine Learning Department, Carnegie Mellon University, Pittsburgh 15213, Pennsylvania, United States

## Abstract

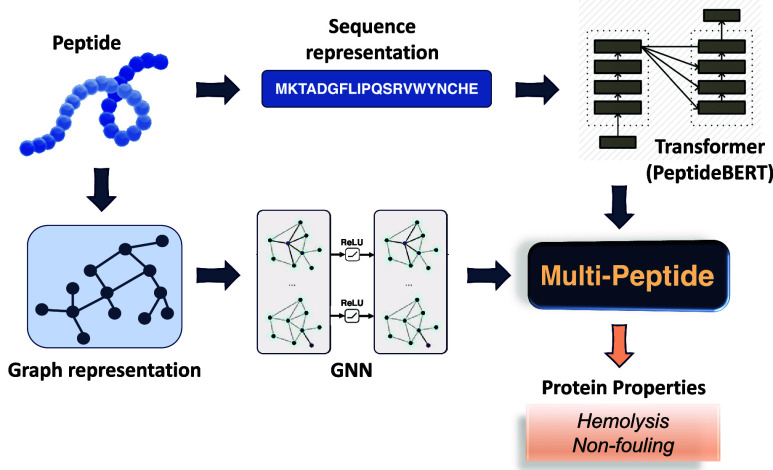

Peptides are crucial in biological processes and therapeutic applications. Given their importance, advancing our ability to predict peptide properties is essential. In this study, we introduce Multi-Peptide, an innovative approach that combines transformer-based language models with graph neural networks (GNNs) to predict peptide properties. We integrate PeptideBERT, a transformer model specifically designed for peptide property prediction, with a GNN encoder to capture both sequence-based and structural features. By employing a contrastive loss framework, Multi-Peptide aligns embeddings from both modalities into a shared latent space, thereby enhancing the transformer model’s predictive accuracy. Evaluations on hemolysis and nonfouling data sets demonstrate Multi-Peptide’s robustness, achieving state-of-the-art 88.057% accuracy in hemolysis prediction. This study highlights the potential of multimodal learning in bioinformatics, paving the way for accurate and reliable predictions in peptide-based research and applications.

## Introduction

Peptides, composed of distinct sequences of amino acid residues, serve as essential components in numerous biological processes and applications.^1−3^ Their distinctive features, such as the potential to cause hemolysis or exhibit nonfouling behavior, are key factors in the development of peptide-based drugs and biomaterials.^4^ For instance, hemolysis, the process by which red blood cells are ruptured, poses significant challenges in designing safe and effective peptide therapeutics.^5^ On the other hand, peptides with nonfouling characteristics, which resist interactions with other molecules, are highly valued in various biomedical contexts.^6^ The functional and structural characteristics of peptides, influenced by their specific amino acid sequences and overall length, determine their interactions with biological systems and the surrounding environment.^7,8^ Understanding the complexity of these interactions is essential for designing customized biomaterials and developing innovative therapeutic strategies.^9−11^

Historically, computational tools like quantitative structure–activity relationship (QSAR) models have been employed to link peptide sequences to their structural characteristics.^12^ However, such methods face challenges in scalability and computational efficiency, particularly when dealing with exponentially expanding sequence repositories.^13^ This limitation underscores the urgent need for innovative computational approaches to decipher the complex association of protein sequences with the corresponding protein properties.

In recent years, machine learning techniques have significantly transformed these methodologies, offering advanced capabilities for data analysis and prediction.^14−18^ Machine learning models are uniquely suited to leverage vast amounts of biological data. The exponential growth in biological data, including protein sequence data through continuous additions to the Protein Data Bank, propelled by advancements in high-throughput sequencing technologies, presents lots of opportunities for predictive modeling of protein properties.^19,20^ In addition to the growth of the protein sequence databases, the advent of advanced protein structure modeling systems such as Google DeepMind’s AlphaFold has significantly accelerated efforts to understand the relationship between protein structure and its functional properties.^21^ AlphaFold, which leverages deep learning techniques to predict a protein’s detailed 3D architecture from just its amino acid sequence, has enabled researchers to bridge the longstanding knowledge gap between sequence and structure in molecular biology.^22^ The widespread availability of fairly accurately generated protein structures now enables researchers to integrate comprehensive structural information into predictive modeling, moving beyond the constraints of relying solely on sequence information.

Deep learning, particularly transformers and large language models (LLMs), has heralded a transformative era in protein structure prediction, providing data-driven insights into the interactions among constituent amino acids.^23−25^ While previous work like PeptideBERT^26^ excels at understanding protein properties based on just their sequences, they may lack the ability to directly incorporate spatial arrangements and interactions among amino acids within the protein structure. The incorporation of another modality, such as protein structural information, is expected to improve the model’s understanding, thereby enhancing the predictive modeling capacity. Multimodal architectures that leverage graph model-based learning have proven to be successful in improving the predictive accuracy across various domains.^28−30,34^ By leveraging Graph Neural Networks (GNNs)^31^ to encode the three-dimensional structure of peptides, we can capture local interactions, spatial arrangements, and other structural features that are not explicitly represented in the sequence data alone. Since peptide property prediction is an important task to configure applications of peptides based on their exhibitional properties, leveraging multimodal training and prediction is expected to improve the current status of the task.

**Figure 1:**
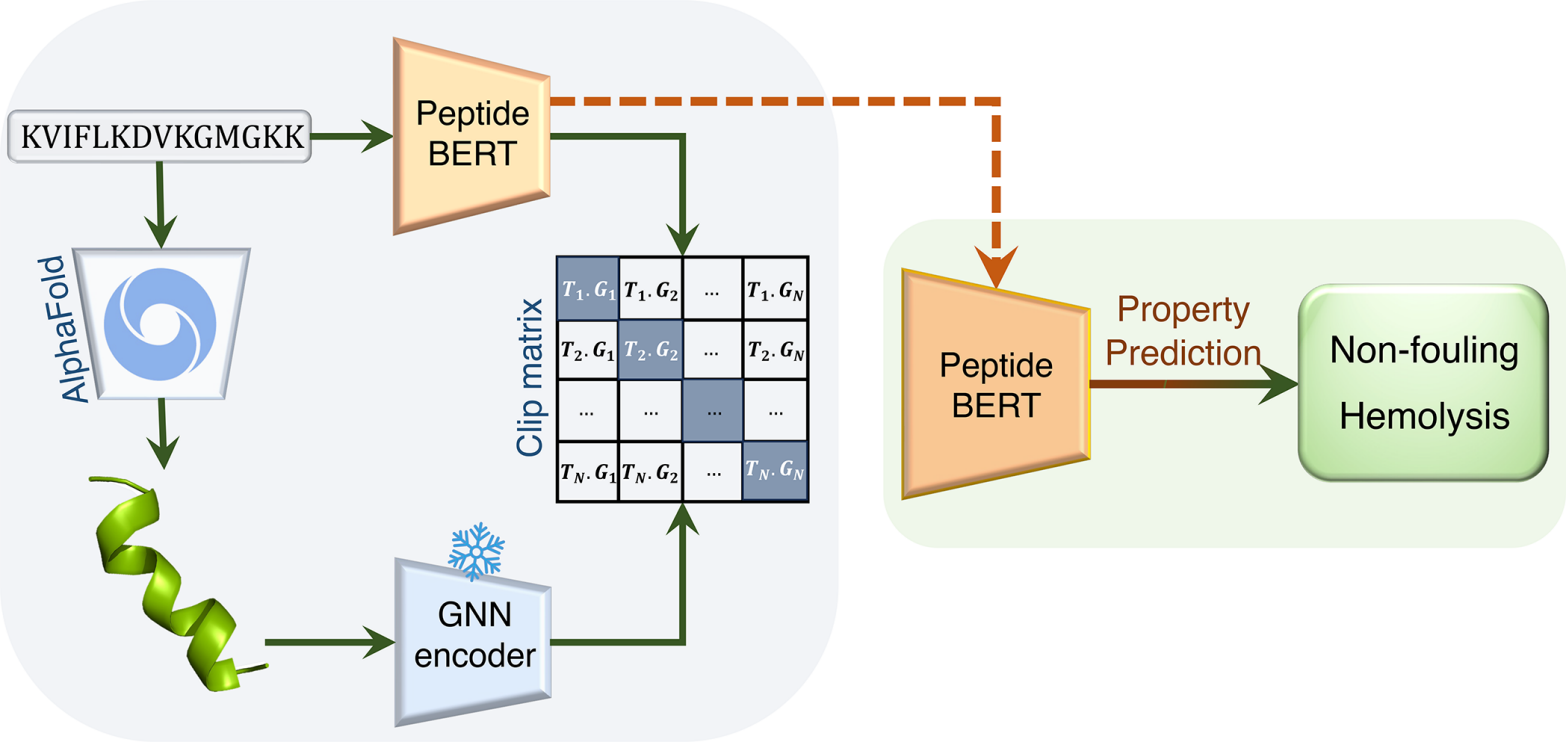
Representation of the Multi-Peptide framework. This figure shows the multimodality-leveraged pretraining through the contrastive loss framework. The GNN weights are frozen to improve the PeptideBERT weights. Inference is done at the end using the updated PeptideBERT weights on each of the test data sets.

In this study, we introduce Multi-Peptide (Figure 1), a multimodality leveraged language-graph learning approach for peptide properties. Multi-Peptide combines PeptideBERT,^26^ a transformer-based language model fine-tuned for peptide property prediction, with a Graph Neural Network (GNN) encoder to learn complex representations of amino acid sequences. We use protein sequences from data sets corresponding to hemolysis and nonfouling behavior^32^ and generate the Protein Data Bank (PDB) files corresponding to each protein sequence using AlphaFold. By pretraining the PeptideBERT transformer model individually on the sequence data and the GNN model individually on the PDB graph data, we take advantage of making each model learn before combining and augmenting their learnings. After initial individual pretraining, we employ a contrastive loss framework, inspired by Contrastive Language Image Pretraining (CLIP),^33,34^ in our ensemble. This enables the PeptideBERT model to learn better, by synergistically combining the global contextual understanding of PeptideBERT with the GNN’s capability to capture local sequence patterns. By leveraging the latent space alignment of the individual model embeddings, this approach represents a promising advancement in peptide property prediction, offering a new methodology for the property prediction of peptides based on both their sequence and derived structure.

## Methods

### Data Sets

**Figure 2:**
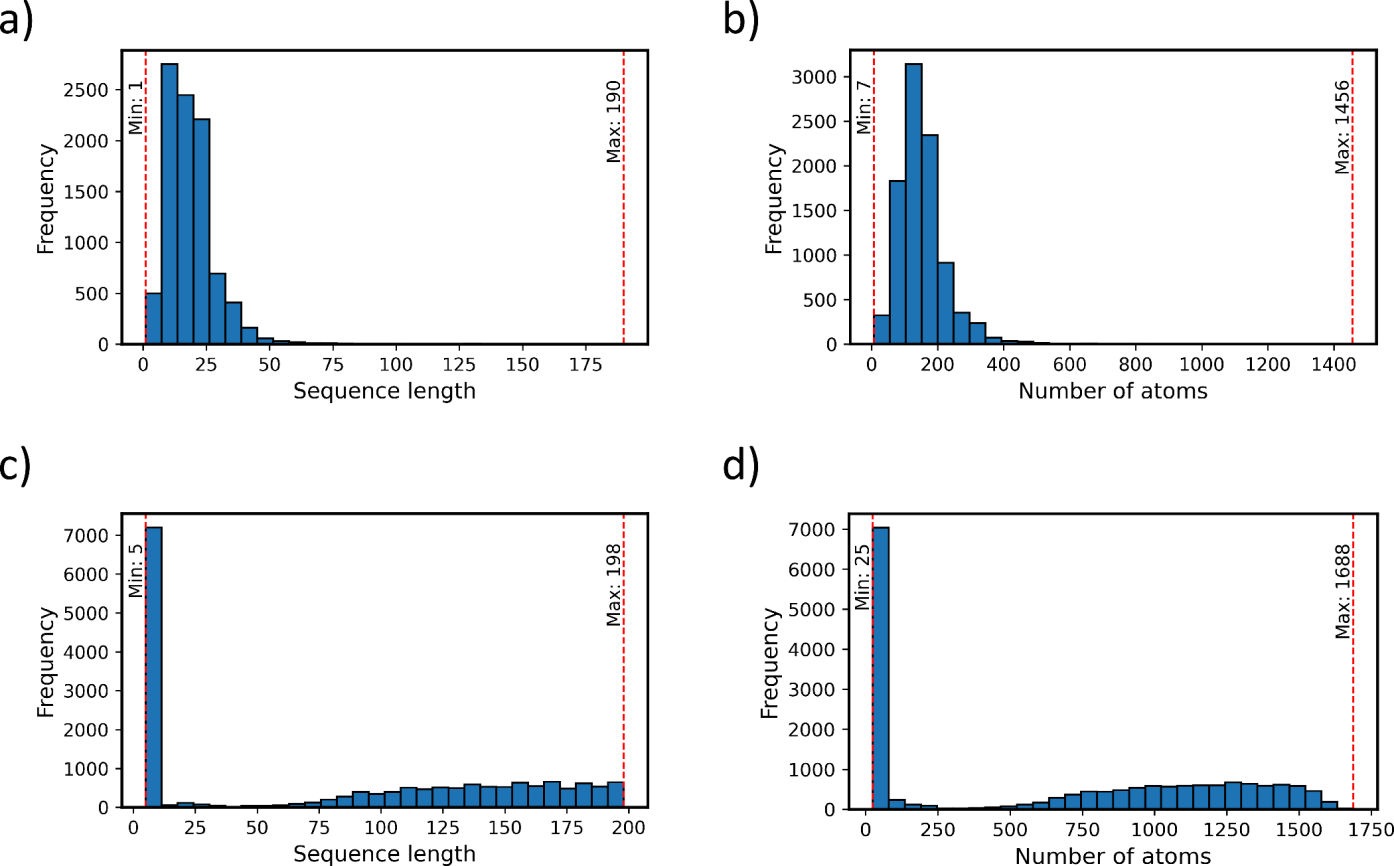
Distribution of peptide sequence lengths across the hemolysis and nonfouling data sets. (a) Sequence lengths vary from 1 to 190 amino acids in the hemolysis data set. The distribution is not uniform, showing a prominent peak and a spread. (b) Number of atoms vary from 7 to 1456 in the hemolysis data set. The distribution mirrors the corresponding sequence length distribution. (c) Sequence lengths range from 5 to 198 amino acids in the nonfouling data set. The distribution is relatively uniform at higher lengths, with a peak at lower values and sparse occurrences over a wide range. (d) Number of atoms vary from 25 to 1688 in the nonfouling data set. The distribution mirrors the corresponding sequence length distribution.

The data sets for hemolysis and nonfouling behavior consist of protein sequences paired with labels indicating whether each sequence corresponds positively or negatively to the respective property.^32^ The protein sequences are represented through text as letter sequences and are of various lengths as seen in Figure 2. For this study, the sequence data from the referenced data set is fed into the AlphaFold2 system to gather structural information about the peptides. The output of the AlphaFold2 framework is a Protein Data Bank (PDB) file containing detailed atomic coordinates among other information, which provides crucial insights into the structural arrangement of proteins.

Computational techniques are employed to predict hemolytic properties, utilizing the Database of Antimicrobial Activity and Structure of Peptides (DBAASPv3).^35^ Due to experimental variability, the sequences appear multiple times with different labels in the data set. After removing duplicates with both positive and negative labels, we are left with 845 positively marked sequences (15.065%) and 4764 negatively marked sequences (84.935%).

Information for forecasting resistance against nonspecific interactions (nonfouling) is gathered from a study^36^ and employed on a data set of 3,600 positively marked sequences and a data set of 13,585 negatively marked sequences. Here, being positively marked indicates a nonfouling protein sequence. Removing 7 sequences that were duplicates having the same label, and 3 sequences for which AlphaFold2 failed to generate the corresponding structural PDB files, we are left with 3596 positively marked sequences (20.937%) and 13579 negatively marked sequences (79.063%). Negative examples are derived from insoluble and hemolytic peptides, along with scrambled negatives (with a length similar to the positive sequences), following an approach outlined in a referenced work.^37^

The data sets were preprocessed via a custom encoding method, where each of the 20 amino acids was represented by its corresponding index in a predefined array. To ensure compatibility with our ensemble, we had to ensure that the data could be used by both the PeptideBERT^26^ model and the GNN. For PeptideBERT, which is built on top of ProtBERT,^38^ the data sets were converted back from integers to letter character using reverse mapping and then re-encoded using ProtBERT’s encoding scheme. For the graph neural network (GNN), the input data consisted of features extracted from AlphaFold2-generated PDB files. These features included atom coordinates (x, y, z), atomic number, atomic mass, atomic radius, indication of whether the atom is part of a side chain or backbone, residue index, number of atoms in the residue, and residue sequence number.

The available data sets are imbalanced, with a higher number of negative examples compared to positive examples, due to properties of the naturally occurring peptides. To address this challenge, we employed a balancing technique known as oversampling in which we increase the number of positive examples by duplicating them. This technique ensures that the model is trained on a balanced data set, thereby preventing the model from being biased toward the majority class. We finally split each data set into two nonoverlapping subsets: a training set (to train the model) consisting of 80% of the entire data set, and a test set (to benchmark the model’s generalization performance on unseen data) consisting of the remaining 20% of the data set.

### Model Architecture

In this study, we propose an innovative approach that aims to improve the prediction accuracy of a transformer-based language model for specific peptide properties by leveraging transferable learning from a different modality. We build upon previous work, PeptideBERT,^26^ which is a transformer-based model that predicts peptide properties based on just the sequences, and now introduce protein structure (graphical) data to capture additional dependencies. This introduction of a new modality of data, along with a corresponding model trained on it, helps the predictive capabilities of the transformer model by transferring its learning through the embedding space. The overall model framework for training thereby comprises three key components: the pretrained language model (PeptideBERT), the Graph Neural Network (GNN), and the shared latent space for the contrastive loss computation across the two modalities.

#### PeptideBERT Transformer

We take the transformer-based PeptideBERT^26^ as our base model. PeptideBERT was fine-tuned over a smaller version of the pretrained ProtBERT^38^ to specifically predict peptide properties based on textual peptide sequences. This fine-tuning was achieved by attaching a tunable head to the pretrained ProtBERT model. This transformer-based BERT model^39^ has the attention mechanism at its heart^40^ and processes protein sequences and their corresponding attention masks to generate contextual text embeddings through the underlying encoder. This model is great at capturing long-range dependencies and global context but struggles with capturing local features and other dependencies. Therefore, we aim to leverage an additional mode of data to enhance the predictive capabilities of this framework.

#### GNN Module

The introduction of the peptide-structural data necessitates the use of a corresponding model that deals with the graph-based inputs, in this case, a GNN model. This structural data was generated as PDB files directly from the peptide sequences using AlphaFold, as discussed earlier. Since each amino acid in a peptide will have multiple constituent atoms, the structural data captures all information about all atoms present in the entire sequence. Eleven features (as mentioned earlier) corresponding to each constituent atom are chosen to be the nodes of the graph-based GNN model, and the edges of the graph network represent the relationship between the nodes. The GNN module, leveraging PyTorch Geometric’s SAGEConv layer, conducts graph convolution on protein sequence graphs, aggregating information from neighboring nodes iteratively.^41^ It is followed by a fully connected neural network incorporating rectified linear unit (ReLU) activation functions and a sigmoid layer, to convert the aggregated information into suitable graph embeddings. The GNN implementation over the protein structure data aims to augment the transformer model’s understanding by focusing on local features and structural relationships.

#### Shared Latent Space and Pretraining

Projection heads are used to map the graph and text embeddings into a unified latent space to implement contrastive learning between the two modalities. These projection heads consist of linear projection layers with Gaussian error linear unit (GELU) activation, dropout, and layer normalization. The integration of the graph and language models to have both embeddings onto a shared latent space within a single module facilitates the joint learning of properties from structural and textual representations from protein data.

Our approach leverages pretraining individually for both the PeptideBERT model and the GNN model, which will later be used in the ensemble as above, through contrastive learning over the shared latent space.^42,43^ By leveraging pretrained weights for each model, acquired from training over each of the data sets, our models gain the ability to predict over the specific tasks. Since our aim in this study is to improve the prediction accuracy of the transformer model, we leverage the pretrained GNN model to transfer its knowledge to the transformer through the shared latent space. This process aims to make the transformer model improve its weights, leading to better performance of the transformer model during inference. The pretrained individual models are combined in the same latent space by using a contrastive learning strategy. A variant of the Contrastive Language-Image Pretraining (CLIP) loss is employed in the fine-tuning stage of the targeted task of peptide property prediction.^33^

#### Contrastive Learning

CLIP,^33^ pioneered by OpenAI, enhances the capabilities of pretrained models by enabling them to reason across different data modalities, such as graphs and text. This method uses a technique called contrastive learning, in which the framework is trained to link related pairs of inputs while distinguishing unrelated ones. To be more specific, in the embedding space, we want to learn about pairs of sequences and structures that are genuine matches (i.e., a given structure corresponds to a particular protein sequence), while also learning about all other combinations of sequence-structures to be unrelated.

At its core, the training process relies on a loss function that uses a softmax operation to normalize similarity scores between the embeddings generated by the Graph Neural Network (GNN) and PeptideBERT. These similarity scores, adjusted by a temperature parameter to control their scale, guide the model’s learning. This ensures that embeddings from both modalities align meaningfully, capturing both their semantic and structural relationships. For a particular protein, say *p*, the graph (g) and text (t) embeddings generated by the GNN and PeptideBERT encoders are represented by
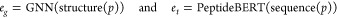
respectively. Let the similarity between two vectors (in this case the two modalities’ embeddings) *x* and *y* be measured and represented through the function sim(*x*, *y*), where the function sim(*x*, *y*) is the dot product between the normalized embeddings, reflecting the cosine similarity.

The loss calculation involves using cross-entropy loss to compare the predicted similarities between the embeddings and their target values. These predicted similarities are computed as the dot product between embeddings from PeptideBERT and the transposed embeddings from the GNN. By optimizing this loss, the model learns to link relevant textual data (protein sequences) with their corresponding graphical representations (protein structures) while “pushing away” the irrelevant pairs.

Mathematically, the overall symmetric loss, *L*(g, t), between the graph and text modalities (denoted by g and t, respectively) is given by
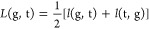
where
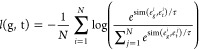
and the temperature parameter of the analysis is denoted by τ.

The objective of the contrastive loss in this binary classification task is to train a model to accurately discriminate between two classes (e.g., positive and negative) by learning representations that effectively capture the semantic and graphical content of the data. The contrastive loss encourages the model to assign distinct and discriminative representations to positive and negative examples, facilitating a better separation between the two classes.

#### Weight Updates and Inference

In our study, we backpropagate through the contrastive loss matrix after pretraining each model (PeptideBERT and GNN) individually over each protein property data set. We freeze the pretrained GNN model’s weights during backpropagation, allowing the algorithm to update the PeptideBERT model’s weights. After the PeptideBERT weights are updated, we use it as a zero-shot classifier for these specific datasets during inference of unseen peptide sequences. Here, it is important to note that the GNN model was used only to improve the PeptideBERT weights and that the graph model is discarded at inference time. Therefore, by leveraging pretrained individual models to incorporate multimodality in training using CLIP loss, the PeptideBERT model gains a deeper understanding of the relationships between peptide sequences and the corresponding properties.

## Results and Discussion

### Training Strategy

The PeptideBERT model and the GNN model were each trained individually on both of the data sets. The specific parameters in each of the model architectures and the relevant hyperparameters used during pretraining are shown in Supporting Information. These hyperparameters, along with the GNN architecture, were chosen to give the best performance.

The individually pretrained models were projected onto the same latent space with contrastive loss implemented. The contrastive loss-implemented ensemble was trained for 100 epochs; this fine-tuning process was longer than the individual pretraining processes, which had 50 epochs each. The reasoning behind fine-tuning for longer was to ensure that for the given model complexity and data dependence, the model could effectively learn by contrasting the different modalities.

Since the objective of contrastive training is for the model to quickly learn the distinguishable features of the pairs, we employ a higher learning rate of 6.0e-5 (higher than that of the pretraining stage of the transformer). The learning rate scheduler utilized was the Learning Rate on Plateau, used to reduce the learning rate by a factor of 0.4 for a period of 5 epochs. These parameter settings were chosen and fine-tuned through iterative experimentation to maximize the model’s learning over the targeted prediction task, by looking at the training loss curves.

For each task, a separate model was trained on the corresponding data set, with a batch size of 20. The models were trained by using the AdamW optimizer, and the binary cross-entropy loss function was employed within the larger contrastive loss. Training was performed on four NVIDIA GeForce RTX 2080Ti GPUs with 11GiB of memory each.

Once the ensemble is trained, the weights from the contrastive loss matrix are extracted. The weights corresponding to the BERT transformer model are then used for inference on each of the test data sets. We expect the transformer model to have learned synergistically with the GNN model, capturing dependencies based on the features extracted and correlations learned.

### Accuracy Analysis

We first benchmark the individual components, i.e., the individual PeptideBERT and GNN model accuracies, to evaluate our Multi-Peptide architecture against. This benchmark was done on the pretrained models, which were then directly used in the multimodal training, so they provide a method to fairly evaluate the effectiveness of the method. As seen in Table 1, the improvement in Multi-Peptide’s accuracy for the Hemolysis data set even though the individual components have lower accuracy individually, demonstrates the capability of the contrastive loss-implemented ensemble to learn from the presence of multiple modalities. However, we observe that the nonfouling data set’s accuracy of the Multi-Peptide framework has dropped when compared to the PeptideBERT accuracy; this could be due to improper learning across modalities, which is discussed later in this study.

**Table 1:** Accuracy of the multimodal framework along with the individual components

Data set	Model	Accuracy (%)
Hemolysis	Multi-Peptide’s BERT (this study)	**88.057**
	Pretrained PeptideBERT	85.981
	Pretrained GNN	83.24
Nonfouling	Multi-Peptide’s BERT (this study)	83.847
	Pretrained PeptideBERT	**88.150**
	Pretrained GNN	79.42

Additionally, as seen in Table 2, the accuracy of the multimodality-leveraged transformer being on par with a fine-tuned PeptideBERT and other models,^26,44,45^ demonstrated the advantages of the introducing and leveraging an additional mode of data. While PeptideBERT excels in the specific property prediction task due to its fine-tuning, our study highlights the robustness and capability of Multi-Peptide to predict protein properties by integrating both sequence and structural data. Multi-Peptide’s BERT gives state-of-the-art (SOTA) results for the Hemolysis data set in particular.

**Table 2:** Accuracies of models on different datasets

Data set	Model	Accuracy (%)
Hemolysis	Multi-Peptide’s BERT (this study)	**88.057**
	Fine-tuned PeptideBERT^26^	86.051
	HAPPENN^44^	85.7
Nonfouling	Multi-Peptide’s BERT (this study)	83.847
	Fine-tuned PeptideBERT^26^	**88.365**
	Embedding + LSTM^26^	82.0
	One-hots + RNN^45^	76.0

In this particular analysis, the accuracy of Multi-Peptide’s ensemble does not surpass that of the PeptideBERT for the Nonfouling data set. This may be due to several factors.

From Table 1, we see that the GNN accuracy is comparatively lower than the PeptideBERT accuracy, showing that the GNN model does not learn as well as the PeptideBERT transformer. This naturally means that the structure representations might not be as correlated to the nonfouling property as the sequences are, indicating that the prediction of the nonfouling properties through structures is inherently a more difficult task than the prediction from sequences. While this goes against the current understanding of protein property prediction,^46^ we aim to understand the discrepancy from an embedding space point of view later in this study.

To address the challenge of lower accuracy, it is also critical to examine both the data quality and model integration. The effectiveness of the GNN relies heavily on the AlphaFold model’s capability to generate highly accurate protein structure data. However, protein structure data can be a lot noisier as compared to sequence data.^47^ Incorporating noisy data can degrade the overall model performance. In an attempt to mitigate this, we have generated 5 PDB files for each sequence and chosen the PDB file with the highest confidence score to represent the structure of the protein. The confidence score, measured through pLDDT, gives an idea of which part of the AlphaFold predicted structure maybe accurate and which part may not.^46,48^ An overall pLDDT score of above 70 corresponds to a decently predicted protein backbone, which is typically observed in the chosen PDB structure having the highest confidence score. Regions where the score is less than 70 typically have inaccurate structure predictions, which might have a direct negative correlation on the property prediction task.

Additionally, combining sequence-based features from the transformer with structure-based features from the GNN presents challenges in effectively aligning these different feature types. If the features are not well-aligned or complementary, the ensemble struggles to learn useful representations. Introducing a GNN model into the ensemble significantly increases overall complexity, necessitating more extensive and precise data for effective training compared to the sequence-based transformer model.

### Embedding Space Analysis for the Nonfouling Data Set

**Figure 3:**
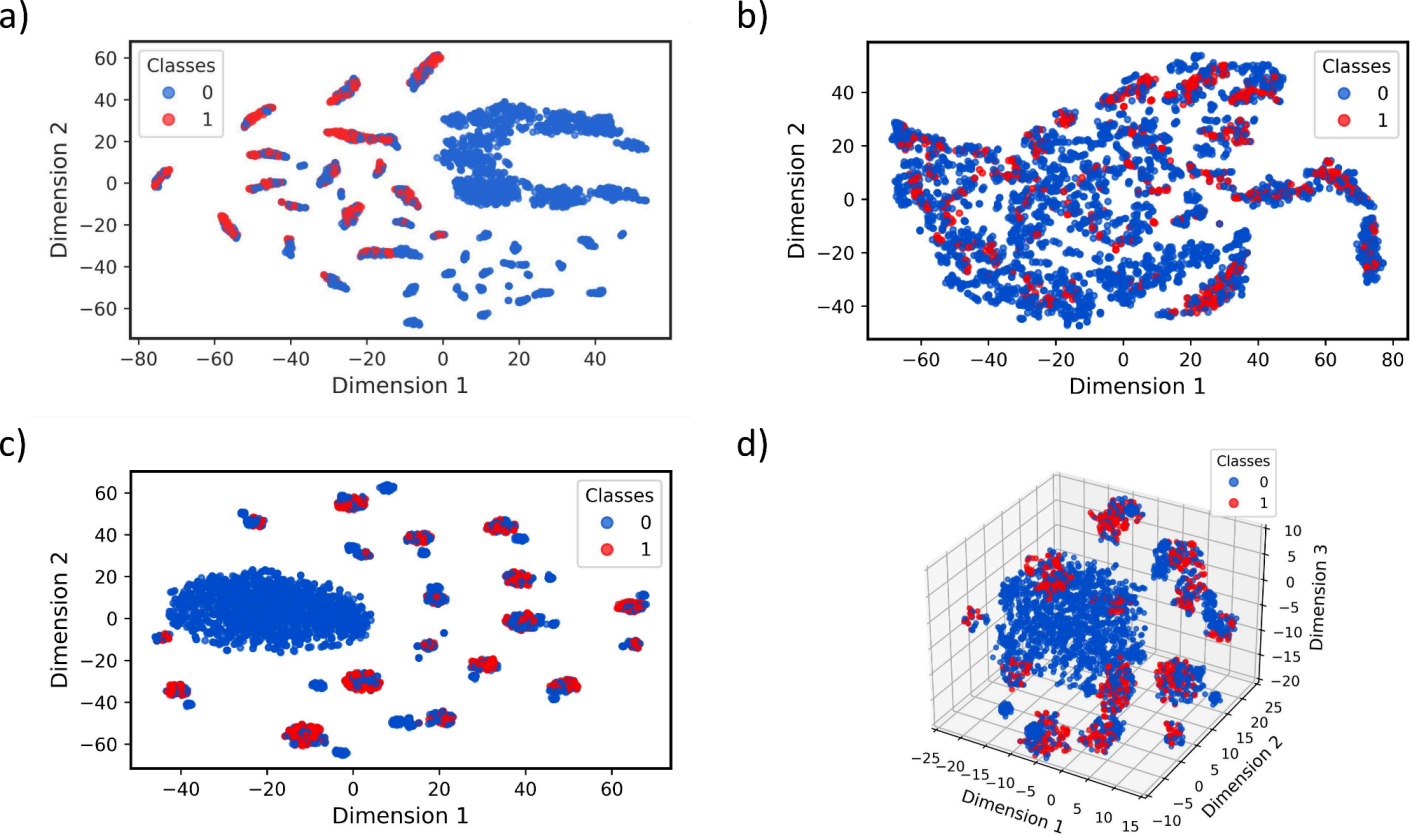
tSNE plots for the embeddings corresponding to the nonfouling data set. The blue points represent the negatively marked sequences, while the red points denote the positives. (a) 2D tSNE plot of the embedding space generated by the PeptideBERT encoder. (b) 2D tSNE plot of the embedding space generated by the GNN encoder. (c) 2D tSNE plot of the shared latent space after CLIP. (d) 3D tSNE plot for the shared latent space after CLIP.

To further explain the performance and capabilities of our models, we employ t-distributed stochastic neighbor encoding (t-SNE) to visualize the embedding spaces derived from various methodologies. t-SNE is a powerful tool for dimensionality reduction that helps in understanding the structure of high-dimensional data by projecting it into a lower-dimensional space.^49^ By visualizing these embeddings, as seen in Figure 3, we aim to provide a qualitative analysis of how well each model captures the underlying patterns and relationships within the peptide sequences. This visualization will help us understand the clustering behavior and the degree of separation between different classes, which are crucial for the accuracy and effectiveness of the models. In this study, we utilized data sets with a significant class imbalance, with a notably larger number of negatively marked sequences compared to positively marked ones. The t-SNE visualizations for PeptideBERT, GNN, and postcontrastive loss embeddings offer critical insights into how each model handles this imbalance and achieves class separation. In particular, this section aims to qualitatively explain the contrastive loss embedding process for the nonfouling data set to understand if the multimodal pretraining process enhances the separation of classes, since the test accuracy seems to be slightly lower than that of the fine-tuned PeptideBERT model.

The t-SNE plot of PeptideBERT embeddings revealed a central cluster of negatively marked sequences surrounded by smaller, dispersed groups containing both negatively and positively marked sequences. This indicated that the PeptideBERT model not only captures semantic similarities within each class but also shows some overlap between classes, highlighting the challenge of clear class separation with the BERT architecture alone.

Contrarily, the t-SNE plot of GNN embeddings demonstrated a more segregated distribution. While a significant pattern of negatively marked sequences occupied the majority of the plot, the positives mirrored a similar pattern with very minimal segregation. The overlap between classes indicates that the GNN struggles to achieve clear class separation, despite its potential to encode structural relationships within peptide sequences. This suggests that while the GNN captures structural nuances, its ability to form distinct class-specific clusters remains limited for this data set. This was reflected earlier in the accuracy analysis, where the GNN’s accuracy was comparatively lower than the PeptideBERT model for the nonfouling data set.

Following multimodality-leveraged (graph-assisted) pretraining, the t-SNE plots showed a prominent central cluster of negatively marked sequences, surrounded by smaller, more concentrated patches of mixed classes. These mixed patches appeared smaller compared to the PeptideBERT embeddings, suggesting that multimodal pretraining was effective in contrasting the classes in the reduced-dimensional space. This is more prominent in the 3D t-SNE plot following the contrastive loss implementation, where we can observe some separation within the smaller mixed clusters in the 3D space, as well.

The t-SNE visualizations provide valuable insights into the strengths and limitations of each embedding technique for peptide sequence classification. Through the embedding space, we observe that PeptideBERT demonstrated semantic understanding, GNN tried to capture structural relationships but did not succeed as much for this data set, and the contrastive framework attempted to enable improved class discrimination. If the GNN provided a better separation of classes, the contrastive framework would have encouraged class separation even further. These visualizations underscore the potential of multimodal pretraining approaches, such as the one proposed in this paper.

## Conclusion

In this study, we introduced Multi-Peptide, a novel approach that leverages multimodality in machine learning to enhance the prediction accuracy of peptide properties. By integrating a graph neural network (GNN) with the transformer-based PeptideBERT model, we aimed to capture both the sequence-based and structural features of peptides. Our approach utilizes a variant of Contrastive Language-Image Pretraining (CLIP) to align the embeddings from these two modalities into a shared latent space, thereby facilitating more robust predictions.

The results from our experiments demonstrate the potential of Multi-Peptide in advancing peptide property prediction. Integration of the GNN and transformer-based models allows us to capture a broader range of features. The accuracy of Multi-Peptide was comparable to that of the fine-tuned PeptideBERT, having state-of-the-art accuracy on the Hemolysis dataset, highlighting the robustness of our approach in handling complex data structures and extracting meaningful features from both sequence and structural information.

In conclusion, Multi-Peptide represents a significant step forward in leveraging multimodality for peptide property prediction. Our approach holds promise for enhancing the accuracy and robustness of predictive models, offering a deeper understanding of peptide characteristics. Future work will focus on refining the integration of modalities and further optimizing the model architecture to fully harness the complementary strengths of sequence-based and structural features. This study underscores the importance and potential of multimodal learning in advancing the field of bioinformatics, through Multi-Peptide’s ability to integrate sequence and structural data.

## Data Availability

The necessary code and data used in this study can be accessed here: https://github.com/srivathsanb14/MultiPeptide.

## Supporting Information Available

Detailed GNN architecture specifications, including layer-wise descriptions. Additional tables documenting PeptideBERT and GNN model parameters, individual pretraining hyperparameters, and CLIP process training times across data sets (PDF)
