# Supplementary material for: Multi-Peptide: Multimodality Leveraged Language-Graph Learning of Peptide Properties
Source: J Chem Inf Model. 2024 Dec 19;65(1):83–91. doi: 10.1021/acs.jcim.4c01443 (PMC11733943; doi:10.1021/acs.jcim.4c01443)
Supplement: Supplementary file 1 — ci4c01443_si_001.pdf [file ci4c01443_si_001.pdf]

Supporting Information: Multi-Peptide: Multimodality Leveraged Language-Graph Learning of Peptide Properties

Srivathsan Badrinarayanan,[†] Chakradhar Guntuboina,[‡] Parisa Mollaei,[¶] and Amir Barati Farimani[*,¶,§,†,‖]

†*Department of Chemical Engineering, Carnegie Mellon University, 15213, USA*

‡*Department of Electrical and Computer Engineering, Carnegie Mellon University, 15213, USA*

¶*Department of Mechanical Engineering, Carnegie Mellon University, 15213, USA*

§*Department of Biomedical Engineering, Carnegie Mellon University, 15213, USA*

‖*Machine Learning Department, Carnegie Mellon University, 15213, USA*

E-mail: barati@cmu.edu

GNN model architecture

The Graph Neural Network (GNN) model utilized in our work is a custom implementation based on PyTorch Geometric, specifically leveraging the SAGEConv layer for graph convolution. The architecture is designed to process graph-structured peptide data and predict peptide properties. The key components of the model are:

- Graph Convolution Layer: A single SAGEConv layer with an input dimension of 11 (matching the peptide features) and an output dimension of 128. This layer captures local structural and feature-based relationships between nodes.

- Fully Connected Layer (FC): A sequential network with two hidden layers ($128 \rightarrow 512 \rightarrow 2048$) and non-linear ReLU activations. This part of the network maps the graph convolution outputs to a higher-dimensional latent space, facilitating better projection. The final output of this block is passed through a sigmoid activation function for normalization.

- Global Pooling: The global max pooling operation aggregates information across all nodes in a graph to produce a graph-level embedding, which serves as input to the final prediction layer.

- Classification Head: A fully connected layer designed for classification tasks, with a hidden size of 512 and an output size of 1, with a sigmoid activation.

This specific model architecture was chosen due to the optimal performance on the datasets. Having a more complex architecture led to overfitting and thereby a reduction in performance accuracy on the test set. A weaker model (with smaller embedding dimension) did not capture the relationships enough to have a good enough accuracy. The finalized architecture gave the best performance overall.

Individual model pre-training

Table S1 shows the parameters associated with the individual PeptideBERT and GNN model architectures. These models were used for individual pre-training, and the respective hyperparameters used as also listed in the same table. These hyperparameters were meticulously chosen after thorough analysis. This step of pre-training was done for 50 epochs each, with a batch size of 20. The projection dimension of the GNN was made to be 2048 to enhance model projection and thereby capture more information. The GNN had 11 input features, as listed before. The PeptideBERT configuration included a hidden size of 256, 8 hidden layers, 8 attention heads, and a dropout rate of 0.10, with a vocabulary size of 25.

Table S1: PeptideBERT and GNN model architectures

PeptideBERT Parameter	Value
Number of input parameters	1
Vocabulary size	25
Hidden size	256
Hidden layers	8
Attention heads	8
Dropout	0.10
Learning rate (pre-training)	1e-5
Scheduler factor (pre-training)	0.1
Scheduler patience (pre-training)	6

GNN Parameter	Value
Number of input parameters	11
Hidden dimension	2048
Learning rate (pre-training)	1e-4
Scheduler factor (pre-training)	0.4
Scheduler patience (pre-training)	10

The hyperparameters associated with the CLIP process have been listed in the manuscript. Table S2 shows the CLIP training time for each dataset, thereby showing the associated time complexity. It is important to note that this process is carried out after the individual pre-training step.

Table S2: Training time for the CLIP process for each dataset

Dataset	Training time (minutes)
Hemolysis	59
Non-fouling	194

The individual components of each model are well-studied in this study, with the accuracy of the PeptideBERT model documented in existing literature too.
